# Efficacy, Safety and Feasibility of Superior Vena Cava Isolation in Patients Undergoing Atrial Fibrillation Catheter Ablation: An Up-to-Date Review

**DOI:** 10.3390/biomedicines11041022

**Published:** 2023-03-27

**Authors:** Dimitrios A. Vrachatis, Konstantinos A. Papathanasiou, Charalampos Kossyvakis, Sotiria G. Giotaki, Gerasimos Deftereos, Maria S. Kousta, Konstantinos E. Iliodromitis, Harilaos Bogossian, Dimitrios Avramides, George Giannopoulos, Vaia Lambadiari, Gerasimos Siasos, Theodore G. Papaioannou, Spyridon Deftereos

**Affiliations:** 1 Department of Cardiology, National and Kapodistrian University of Athens, 11527 Athens, Greece; 2 Department of Cardiology, “G. Gennimatas” General Hospital of Athens, 11527 Athens, Greece; 3 Evangelisches Krankenhaus Hagen-Haspe, Clinic for Cardiology and Electrophysiology, 58135 Hagen, Germany; 4 Department of Cardiology, University of Witten/Herdecke, 58455 Witten, Germany; 5 Department of Cardiology, Aristotle University of Thessaloniki, 54642 Thessaloniki, Greece; 6 Department of Internal Medicine, National and Kapodistrian University of Athens, Medical School, Attikon University Hospital, 12462 Athens, Greece; 7 Department of Cardiology, National and Kapodistrian University of Athens, Medical School, Sotiria Chest Disease Hospital, 11527 Athens, Greece; 8 1st Department of Cardiology, National and Kapodistrian University of Athens, 11527 Athens, Greece

**Keywords:** atrial fibrillation, catheter ablation, non-pulmonary vein triggers, superior vena cava isolation, feasibility, efficacy, safety

## Abstract

Pulmonary vein isolation (PVI) is the cornerstone in atrial fibrillation (AF) ablation; yet, the role of arrhythmogenic superior vena cava (SVC) is increasingly recognized and different ablation strategies have been employed in this context. SVC can act as a trigger or perpetuator of AF, and its significance might be more pronounced in patients undergoing repeated ablation. Several cohorts have examined efficacy, safety and feasibility of SVC isolation (SVCI) among AF patients. The majority of these studies explored as-needed SVCI during index PVI, and only a minority of them included repeated ablation subjects and non-radiofrequency energy sources. Studies of heterogeneous design and intent have explored both empiric and as-needed SVCI on top of PVI and reported inconclusive results. These studies have largely failed to demonstrate any clinical benefit in terms of arrhythmia recurrence, although safety and feasibility are undisputable. Mixed population demographics, small number of enrollees and short follow-up are the main limitations. Procedural and safety data are comparable between empiric SVCI and as-needed SVCI, and some studies suggested that empiric SVCI might be associated with reduced AF recurrences in paroxysmal AF patients. Currently, no study has compared different ablation energy sources in the setting of SVCI, and no randomized study has addressed as-needed SVCI on top of PVI. Furthermore, data regarding cryoablation are still in their infancy, and regarding SVCI in patients with cardiac devices more safety and feasibility data are needed. PVI non-responders, patients undergoing repeated ablation and patients with long SVC sleeves could be potential candidates for SVCI, especially via an empiric approach. Although many technical aspects remain unsettled, the major question to answer is which clinical phenotype of AF patients might benefit from SVCI?

## 1. Introduction

The discovery of pulmonary veins (PVs) as an important source of atrial ectopic beats initiating atrial fibrillation (AF) by Haïssaguerre et al. launched a new era in electrophysiology and has literally transformed AF management [1]. Despite the fact that PV isolation (PVI) remains the mainstay in AF ablation, long-term success rates are suboptimal [2]; hence, curative therapies in AF delineate an unmet clinical need.

Non-pulmonary vein foci are quite often involved in AF genesis and superior vena cava (SVC) ectopy is a common culprit [3], especially in young, female subjects with non-paroxysmal AF (NPAF) and biatrial cardiomyopathic substrate [4,5,6]. Approximately one in three persistent AF (peAF) subjects feature non-PV foci, and SVC is quite prevalent (17%) [7].

PV reconnection remains the major source of AF recurrence post-ablation, yet the presence of non-PV foci at the index procedure might be predictive of multiple redo procedures [8]. They are not infrequently encountered even among paroxysmal AF (PAF) subjects, ranging from 6% to 31.5%, depending heavily on the employment or not of provocative protocols in order to unmask an arrhythmogenic SVC [9,10,11,12,13].

The current review aims to describe the different AF ablation approaches pertaining to SVC isolation (SVCI), as well as to summarize their efficacy, feasibility and safety outcomes.

### Superior Vena Cava Arrhythmogenicity and Isolation

Historical animal studies by Zipes et al. [14], Ito et al. [15] and Spach et al. [16] pinpointed the arrhythmogenic properties of the SVC decades ago. Myocardial sleeves are commonly encountered in the SVCs from subjects with and without AF, and most of the time they are heterogeneous and asymmetric [17]. As far as the SVC–right atrial (RA) junction is concerned, the myocardial sleeve transition pattern can be continuous or discontinuous; additionally, longitudinal or circumferential patterns have been described [18].

SVC sleeve length varies widely, ranging from 0.5 to 6 cm, and sleeves harboring ectopic foci are usually longer than 3 cm [19]. Myocardial atrial extensions as well as electrophysiological properties differ between SVC and PV sleeves. SVC sleeves are mainly present on the endocardial side, featuring both increased automaticity and triggered activity, whereas PV sleeves are predominantly epicardial, featuring triggered activity [20,21]. Of note, SVC and right superior PV (RSPV) sleeve length and arrhythmogenicity are positively correlated [22].

The role of SVC in AF pathogenesis is not completely understood. SVC sleeves can act, with a decreasing order of frequency, as a trigger, driver or combination of them in AF subjects [23]. SVC cardiomyocytes are also influenced by autonomic innervation and the adjacent aortocaval ganglion preferentially triggers SVC ectopic beats [24]. SVC foci are usually going unnoticed in the electrophysiology lab, unless several provocative maneuvers (AF cardioversion, adenosine triphosphate and/or isoproterenol (ISP) infusion) are undertaken [9,23]. What is more, SVC foci might feature longer effective refractory intervals and thus can only be revealed after PVI [25].

The usual SVC isolation procedure involves a first step of 3D electroanatomical mapping of the SVC–RA junction and a second step of applying radiofrequency (RF) ablation, just 5–10 mm above the SVC–RA junction in order to avoid complications such sinus node or right phrenic nerve injury, as well as SVC stenosis [3,9,26]. Ideally, SVCI should be performed below the level of the SVC triggers and above the level of the sinus node origin [27]. High output pacing (usually 10–30 mA) is performed on the posterolateral wall of the SVC to identify areas with phrenic nerve capture. Many technical differences exist, such as circumferential versus segmental point by point SVCI, empiric (prophylactic) versus as-needed SVCI (SVC triggers and/or drivers), as well as varying energy sources, ablation protocols and provocative maneuvers.

## 2. Material and Methods

Studies including patients undergoing catheter ablation for AF in whom SVCI was also performed were evaluated for inclusion in this review. Two reviewers (DV, KP) independently identified the relevant studies by an electronic search of the MEDLINE database from inception to 29 December 2022. The following search query was used: “superior vena cava” and “atrial fibrillation ablation”. Articles and book chapters cited in the reference lists of initially identified articles by this query were reviewed in order to identify any supplemental studies.

In order for a study to be eligible it had to fulfill the following inclusion criteria: (1) evaluated AF patients undergoing SVCI, (2) employed a clearly stated description of SVCI protocol and (3) reported data on SVCI efficacy and/or feasibility and/or safety. Studies were excluded if they were (1) case reports, or (2) evaluating non-catheter based ablation modalities for AF (surgical, epicardial or hybrid ablation).

## 3. Results

### 3.1. Randomized Studies and Meta-Analyses

Three randomized controlled trials have compared PVI versus PVI plus empiric SVCI in patients with AF and have found no substantial differences as far as arrhythmia recurrence is concerned (see Table 1). Wang et al. randomized 106 patients with PAF to undergo PVI plus empirical SCVI (circumferential or segmental) or PVI alone. SVC venography was utilized to define SVC–RA junction and high output pacing (30 mA) was performed on the posterolateral wall of the SVC to identify areas of diaphragmatic stimulation. The blanking period in this study was shortened to 1 month and all patients in the SVCI arm underwent computed tomography at 3 months to assess both PV and SVC ostia. SVCI was both feasible (50/52) and safe (no case of sinus node or phrenic nerve injury or SVC stenosis); yet, after 12 months of follow-up, recurrence rates were comparable between the two groups [28]. SVCI was not accomplished in two cases (circumferential approach) because of obviating phrenic nerve capture. In 78% of cases in the SVCI arm, patients underwent segmental SVCI (mean number of segments 3 ± 1, septal, posterior, lateral and anterior segment in a decreasing order of frequency). No SVC reconnection was encountered in redo cases of the SVCI arm. The small number of enrollees and the relatively short follow-up might have underpowered this trial to detect any efficacy differences [28].

Corrado et al. conducted a larger trial including 320 patients with AF and found that empirical SVCI plus PVI is superior to PVI alone in the subgroup of PAF subjects. Although all AF subgroups (paroxysmal, persistent, and permanent) demonstrated numerically lower AF recurrences in the SVCI arm, this was only statistically significant in PAF subgroup analysis (10% vs. 23%; *p* = 0.04). The protocol of this study precluded anti-arrhythmic drug (AAD) administration post-ablation. Intracardiac echocardiography (ICE) was employed to define the SVC–RA junction at the level of the lower border of the right pulmonary artery, and high output pacing (30 mA) was performed on the posterolateral wall of the SVC to identify areas of diaphragmatic stimulation. All patients underwent computed tomography at 3 months to assess PV and SVC ostia. Any arrhythmia recurrence taking place within the first 2 months of ablation (blanking period) was not considered as a procedural failure. In 26 of 160 patients, SVCI was not feasible because of obviating phrenic nerve injury (PNI) (21 cases or 13%) or no SVC signals (5 cases or 3%). No cases of sinus node or phrenic nerve injury or SVC stenosis were reported [29]. RF ablation was carried out with non-irrigated RF catheters.

Da Costa et al. have also compared PVI with PVI plus empiric SVCI and included 100 PAF patients. High output pacing (30 mA) was performed on the posterolateral wall of the SVC to identify areas of diaphragmatic stimulation, and catheter ablation was conducted remotely through a 3D non fluoroscopic mapping system. A two-month blanking period was applied and AADs were continued for at least three months post-ablation. After 18 months of follow-up, they did not find any differences regarding AF recurrence. Of note, segmental SCVI was feasible in all 51 cases with an irrigated magnetic ablation catheter not exceeding a power of 25 Watts; yet, two patients suffered PNI [27]. The small study population and the relatively lenient monitoring protocol over follow-up are the main limitations of this study.

A meta-analysis conducted by Li et al. [including the three above-mentioned trials (n = 526) as well as an observational study by Ejima et al. (n = 186) [30]] suggested that empiric SVCI is associated with increased arrhythmia free survival as compared to PVI in PAF patients. Nevertheless, this finding was mainly driven by the non-randomized study. The heterogeneous ablation strategies among the included studies and the small sample size are important limitations [31].

Taken all together, available data suggest that empiric SVCI does not pose any advantage over lone PVI in AF subjects; although, patients suffering from PAF might be benefited. A future randomized trial should test irrigated contact force sensing RF catheter approaches in larger samples of PAF patients harboring SVC triggers and/or perpetuators at index or even redo procedures.

### 3.2. Cohorts Comparing Different Superior Vena Cava Isolation Approaches

#### 3.2.1. Empiric vs. As-Needed SVCI

There is only one non-randomized study (evaluating 68 PAF patients) comparing as-needed SVCI-only versus PVI plus as-needed SVCI. Researchers employed several provocation maneuvers, including ISP infusion, burst pacing and/or adenosine infusion followed by external cardioversion if AF was successfully induced; study subjects were divided into purely SVC initiating AF and combined PV and SVC initiating AF subgroups. The SVC–RA junction was determined by ICE or SVC venography, and high output pacing was performed on the anterolateral wall of the SVC to identify areas of diaphragmatic stimulation. SVCI was carried out in a circumferential or segmental manner depending on patient pain tolerance and obviating sinus node injury (SNI) or PNI. An irrigated RF catheter was utilized during ablation. After a long follow-up period of more than seven years, the SVCI-only strategy was associated with reduced arrhythmia recurrence. Of note, arrhythmia recurrence rates did not differ among patients undergoing either segmental (n = 50) or circumferential (n = 18) SVCI (*p* = 0.82). Larger SVC diameter was found to be predictive of AF recurrence [hazard ratio (HR) 1.4; 95% confidence intervals (CI) 1.1–1.8; *p* = 0.02]. In patients undergoing SVCI plus PVI, larger left atrium diameter was the sole independent predictor of arrhythmia recurrence. Left atrium and ventricle diameters were larger in PAF patients undergoing SVCI on top of PVI and might have affected outcomes. Nevertheless, in PAF patients with purely SVC initiating episodes of AF, SVCI might be not inferior to PVI plus SVCI [32].

Two studies have compared empiric versus as-needed SVCI in PAF patients undergoing PVI. Although studies’ populations and demographics differed significantly, they both found that empiric SVCI is associated with reduced arrhythmic events as compared to as-needed SVCI. Ejima et al. compared these two approaches in 186 PAF patients at index procedure. The SVC–RA junction was determined by 3D electroanatomic mapping and high output (10 mA) pacing was performed on the posterolateral wall of the SVC to identify areas of diaphragmatic stimulation. An irrigated catheter was utilized for SVCI at a maximum power of 25 W. SVC foci were identified after ISP (10 mcg bolus) with or without adenosine infusion (20 mg bolus). Since empiric SVCI was performed while waiting for PV reconnection testing, Ejima et al. found that empiric SVCI is associated with shorter procedural time and fluoroscopic time as compared to as-needed SVCI [30].

Zhang et al. compared as-needed versus empiric SVCI in 144 PAF patients undergoing a second (redo) PVI procedure. The provocative protocol included programmed electrical stimulation and intravenous injection of ISP (2–4 mg/min), with or without a 20 mg bolus injection of adenosine. An irrigated catheter was utilized for RF ablation delivery at 25 W; the SVC–RA junction was determined by 3D electroanatomic mapping and SVC venography; high output (20 mA) pacing was performed on the posterolateral wall of the SVC to identify areas of diaphragmatic stimulation; and a segmental or circumferential approach was employed depending on underling heart rhythm, sinus node or AF, respectively. The researchers found that procedural and fluoroscopic time are comparable between the two approaches, while empiric SVCI is predictive of increased arrhythmia free survival [33].

The above-mentioned studies suggest that empiric SVCI is at least as safe and effective as conventional SVCI (as-needed). Procedural data as well as adverse events are also comparable between these two approaches, provided that standard precautions are followed to prevent SNI/PNI, and that empiric SVCI is carried out while watchful waiting for PV reconnection.

#### 3.2.2. As-Needed SVCI on Top of PVI

Only a few cohorts have compared as-needed SVCI plus PVI versus PVI alone. Higuchi et al. have assessed 60 patients with PAF or peAF for SVC initiating AF. Their AF provocation protocol included burst pacing with ISP infusion (0.5–2 mcg/min/kg), followed by internal cardioversion, and if SVC ectopic beats were noted, focal or segmental SVCI was pursued with a 4 mm non-irrigated RF catheter at 20–25 W for 40–60 s. Recurrence rates were comparable between the two study arms; yet, longer (>3 cm) SVC sleeves with larger potentials (>1 mV) are associated with SCV triggers among AF patients [26].

The same group of researchers employed, for the first time, an electrical stimulation provocative protocol and found that SVC acts as a driver of AF in approximately 20% (7/36) of redo ablation subjects. The SVC sleeve length was longer than 3 cm in all patients harboring SVC drivers. Over a three year follow-up period, no case of SVCI showed arrhythmia recurrence; although, the very small number of patients included should be noted [19].

Takigawa et al. have examined 865 PAF patients undergoing three different AF ablation approaches (PVI only, PVI plus as-needed SVCI, and PVI plus as-needed non-PV foci). Their provocation protocol included ISP (5–20 mcg/min) infusion, followed by burst pacing and interval cardioversion. A non-irrigated, 8 mm-tip, RF ablation catheter was utilized. Female gender was predictive of SCV initiating AF. Arrhythmia free survival, as well as adverse events, were comparable between PVI and PVI plus SVCI subgroups. Of note, SVC foci not previously identified were revealed in 25% of PVI only subgroup patients undergoing redo ablation, and SVC reconnection was demonstrated in 53% of the PVI plus SVCI patients undergoing redo ablation [11].

Yoshida et al. targeted spontaneous or ISP infusion (5–20 mcg/min) induced SVC ectopic beats in 22 out of 121 PAF patients and observed that these patients ultimately needed a statistically higher number of redo procedures over a two-year follow-up period. SVC reconnection was reported in 27% of the patients with AF recurrence. Of note, dormant RSPV had a negative predictive value of 95% in ruling out SVC initiating AF [34].

As far as arrhythmia recurrence is concerned, the above-mentioned studies largely failed to demonstrate any clinical benefit of as-needed SVCI in AF patients; although, both safety and feasibility are consistent findings (see Table 2). Patients’ characteristics (mixed populations of PAF and NPAF subjects, index and redo procedures), varying anti-arrhythmic drug administration and monitoring protocols post-ablation, as well as the small number of enrollees, might have underpowered these studies to prove any benefit.

#### 3.2.3. Empiric SVCI on Top of PVI

Three methodologically different studies assessed empiric SVCI in addition to PVI in PAF and peAF subjects and reported inconclusive results. Yoshiga et al. found that empiric segmental SVCI is associated with numerically less arrhythmic events in peAF cases that underwent redo AF ablation and featured no PV reconnection. Regardless of ablation strategy (PVI with or without SVCI), the duration of peAF (>3 years) was the sole factor that predicted AF recurrence among AF patients in need of redo procedures [35].

Knecht et al. compared empiric circumferential SVCI in addition to PVI (if the number of reconnected PVs during redo ablation procedure were ≤1) and found that empiric SVCI does not confer any advantage as far AF recurrences are concerned [36].

Jin et al. carried out empiric linear ablation from the SVC to the RA septum on top of PVI in patients suffering either PAF or PeAF, and found that empiric linear SVCI is associated with increased arrhythmia free survival (HR = 0.59; 95% CI: 0.44–0.78; *p* < 0.001) irrespective of age, comorbidities, gender and AF subtype. Procedure time was longer in the SCVI arm and adverse effects were comparable [37].

Unfortunately, the above-stated cohorts are not comparable owing to the fact that strikingly different SVCI approaches were followed (segmental, circumferential, and septal linear) on top of dissimilar participant characteristics.

The only comparative cryoablation (CBA) cohort in the field is quite encouraging. Overeinder et al. explored right jugular/subclavian empiric SVCI via a second generation CBA catheter and found that this strategy on top of CBA PVI is associated with reduced arrhythmia recurrence over a 12-month follow-up period and slightly increased procedural time as compared to CBA PVI alone among PAF patients [38]. Larger cohorts and randomized trials are needed to validate these findings.

Two additional studies deserve to be mentioned, due to the population studied or the ablation protocol employed by the researchers. First, Kataoka et al. have examined the safety and feasibility of empiric segmental SVCI in patients with cardiac implantable electronic devices (CIED). In an age and sex matched analysis of 68 AF patients, they found that arrhythmia recurrence, adverse effects and procedural aspects such as total procedure time and SVCI time were comparable between CIED and non-CIED arms. Pacing threshold, sensing and lead impedance were not affected post SVCI. Interestingly, SVC reconnection was found in 9 of 12 patients with AF recurrence in the CIED group, and the SVC segment (usually lateral) adjacent to the lead was implicated in all of these cases and was successfully re-isolated in 7 out of the 9 patients [39].

Second, Simu et al. have explored empirical segmental SVCI (using high power short duration RF) on top of PVI plus left atrium substrate modification (roof line, mitral isthmus line, box lesion) in AF subjects undergoing redo procedures, and found no benefit concerning arrhythmia recurrence as compared to PVI plus left atrium substrate modification [40].

### 3.3. Other Non-Comparative Cohorts Reporting on Superior Vena Cava Isolation

Several studies of relatively small numbers of AF subjects and without comparative design have examined the efficacy, safety and feasibility of SVCI. The majority of these cohorts explored as-needed SVCI during index PVI. Only a minority of them included redo AF patients and/or non-RF energy sources. The main findings of these studies are presented in Supplementary Table S1.

Tsai et al. found that SVC initiates AF in 6% of PAF patients and could be effectively and safely ablated with a mean number of five RF applications [3]. Nyuta et al. suggested that SCV length greater than 37 mm and SVC diameter greater than 17 mm could be predictive of SVC foci in AF subjects undergoing PVI [41].

Arruda et al. studied both as-needed SVCI and empiric SVCI on top of PVI in a mixed AF population. They found that 12.6% of AF patients had SVC triggers and all of these patients also featured an arrhythmogenic RSPV. After as-needed SVCI, no AF recurrence and no SVC stenosis (SVCS) were noted. Additionally, empiric SCVI was theoretically feasible in 96% of cases (208/217 patients demonstrated SVC potentials). Ultimately, 59% of AF subjects were treated with segmental ablation and 19% necessitated circumferential isolation, while 18% featured an obviating phrenic nerve capture. High output pacing (30 mA) was effective in reducing PNI cases to zero; yet, it precluded SCVI in 18% of the cases [42]. Miyazaki et al. explored the feasibility of monitoring right phrenic diaphragmatic compound motor action potentials (CMAPs) during segmental SVCI and found that SVCI can be completed without complications in all these cases. However, phrenic nerve capture was observed in 20% of the RF applications and only 1.4% of applications were associated with decreased diaphragmatic CMAPs without developing PNI, thus putting the clinical utility of electromyography-guided phrenic nerve monitoring under question [43].

Chen et al. underlined that SNI should not go unnoticed during SVCI, since it occurs in 4.5% of cases. They also suggested that in order to avoid SNI, high density 3D electroanatomic mapping or usage of ICE to define the SVC–RA junction, as well as segmental ablation of anteroseptal SVC and a higher level of ablation at the anterolateral wall should be adopted. Lastly, SCVI during sinus rhythm is also advisable [44].

Tanaka et al. have studied SVC foci in 113 AF patients with a high resolution 3D mapping system so as to assess the feasibility and safety of circumferential SVCI along a conduction block line [45]. They found that a conduction block is present in approximately one out of two patients undergoing index PVI, featuring a varying length and shape. Yet, more importantly, a diagonal conduction block line traversing from the lower lateral wall to the higher anterior SVC wall is always present in these patients. Phrenic nerve capture occurred more commonly in the non-block group (16/21) as compared to block group (5/22) and RF application times, SVCI time as well as RF energy required were significantly reduced in cases featuring SVC block line. Of note, older patients were more likely to harbor a conduction block line between RA and SVC.

Miyazaki et al. have also utilized a high resolution mapping system and found that the length of the SVC sleeve is asymmetric and variable in both AF and non-AF subjects. Furthermore, the SVC isolation line has a median length of 20.0 mm and a conduction block is present in 8.8% of AF patients undergoing as-needed segmental SVCI, which resulted in fewer RF applications [46].

Gianni et al. have assessed the safety and feasibility of a novel segmental SVCI strategy in order to avoid PNI. They started empiric isolation of the SVC from the septal wall and continued posteriorly and inferiorly towards the RA as guided by early activation mapping. SVCI was feasible in 98%. Nevertheless, they reported two cases of transient PNI and SVC reconnection in 5/13 patients that underwent repeated ablation [47].

Nishiyama et al. have utilized programmed pacing from the right atrial appendage (RAA) to separate SVC and RA potentials and thus construct a 3D map of an electrophysiologically defined SVC–RA junction. A mean number of 9.3 ± 2.0 points were needed, and anterior SVC segment points were higher than posterior points. Three-dimensional maps of phrenic nerve capture were also constructed in 11/15 patients and the following SVC walls were involved in a decreasing order of frequency lateral, posterolateral and posterior [48].

Not infrequently, RA and SVC potentials are tightly packed, hindering the identification of the optimal ablation sites. Fukumoto et al. have found that decremental conduction via the RA–SVC junction is feasible and easily induced by a single extrastimulus from the RAA. They also showed that 3D activation mapping during sinus rhythm facilitates SN localization. Segmental SVCI was feasible in 68%, while the remaining patients required a circumferential approach [49].

### 3.4. SVC Foci and Reconnection during Repeated AF Ablation

Among patients that have previously undergone SVCI, SVC reconnection is common (74%) and the most common segment involved is the anterolateral wall. Successful re-isolation is feasible in 96% with a mean number of 4.2 ± 2.9 RF applications [50]. Accordingly, Miyazaki et al. supported that SVC is implicated as a trigger or driver of AF in 44 out of 836 AF patients (5.3%). Arrhythmogenic SVC could be identified spontaneously or after ISP infusion, after adenosine infusion, or after internal cardioversion in 56.8%, 29.5% and the 13.6% of the cases, respectively. As-needed SVCI was required in 42.1% of cases during the first redo procedure and in half of the cases during the second redo. SVC reconnection was observed in 75% and 100%, respectively [51].

Inada et al. found that lower BMI values and repeated ablation are predictive of SVC foci among PAF patients. SVC is implicated in AF initiation and maintenance in 12.8% of the AF subjects and more than half of the cases (58.1%) were solely identified during redo ablation. Furthermore, patients with SVC foci are more likely to harbor other non-PV foci as compared to patients without SVC foci [52]. Omuro et al. explored empiric SVCI on top of PVI in non PAF patients. They found that an arrhythmogenic SVC was implicated in 33.3%. During redo ablation, PV reconnection was found in 68.3% and SVC reconnection in 82.9% [53].

Takamiya et al. sought the incidence of non-PV foci among PAF patients that have previously undergone CBA. Almost half (48%) of the included patients demonstrated non-PV foci; one out three patients (37%) exhibited non-PV foci for the first time at the redo procedure and the vast majority (80%) featured different non-PV foci from the initial procedure. In addition, non-PV foci were more common among PAF subjects without PV reconnection. SVC was the most prevalent culprit and was implicated in 26%. Non-PV non SVC triggers were also predictive of AF recurrence [54].

Empiric SVCI might also be proven effective in PVI non responders. Gu et al. have examined data from patients that have had AF recurrence and no PV reconnection during redo ablation, and found that empiric SVCI was associated with increased arrhythmia free survival (79.3 vs. 50.0%; HR: 0.338; 95% CI: 0.131–0.873; *p* = 0.025). This was a small retrospective study and larger randomized trials are much anticipated [55].

The previously discussed studies are further illuminating the complex role of SVC in triggering and perpetuating AF among AF patients with arrhythmia recurrence post-ablation. The role of SVCI might be crucial among the so-called PVI non-responders and CBA treated subjects; yet, adequately designed and powered studies are still lacking.

### 3.5. Non RF Ablation Sources

Wei et al. explored the efficacy and safety of as-needed single shot circumferential SCVI through a second generation CBA catheter [56]. CBA of SVC was feasible in 80% of PAF subjects, and the remaining patients needed RF touch up ablation due to an obviating PNI. Regarding procedural data, fluoroscopy time did not differ between patients undergoing as-needed SVCI and PVI only (16.3 ± 9.9 vs. 15.2 ± 5.4 min; *p* =0.546); yet, a longer procedure time was observed in the SVCI subgroup (61.6 ± 15.3 vs. 53.2 ± 9.7 min; *p* < 0.001). SNI and PNI were observed in 7.7% and 19.2%, respectively, yet they were transient and restored during the procedure. Rubio Campal et al. have assessed the safety and feasibility of empiric SVCI via a third generation CB catheter, and after a relatively short follow-up period (<12 month) they reported arrhythmia recurrence in only 11% of the cases [57].

Arceluz et al. explored empiric circumferential laser ablation of SVC on top of PVI and reported a high incidence of PNI 23% (3/13) [58]. AF patients undergoing hot balloon ablation (HBA) demonstrate similar long term recurrence rate as compared to CBA treated controls [59], and HBA has been associated with less PNI as compared to CBA during RSPV isolation [60]. Yet, to the best of our knowledge, no large cohort has explored HBA for SVCI to date. Since CBA and HBA approaches are rapidly expanding worldwide, more safety and efficacy data are bound to emerge in the following years, and large studies of comparative design are needed.

## 4. Summary and Perspectives

SVC can act as a trigger or perpetuator of AF, and its significance might be more pronounced in patients undergoing repeated ablation [51,53]. Moreover, SVC myocardial sleeve length and SVC luminal area have been associated with SVC ectopic activity and arrhythmia recurrence in patients undergoing AF catheter ablation, with an optimal cut-off value of 2.56–2.59 cm^2^ [61]. The current literature supports that discovering arrhythmogenic SVC is highly dependent on the provocative protocol employed [30,33,53].

Many patients with SVC foci are only discovered during redo ablation; hence, finding which clinical phenotype is benefited remains to be answered. ’’The increasing implementation of CBA and contact force RFA might lead to an era of reduced PV reconnection and the number of the so-called PVI non responders necessitating specialized treatment during redo ablation will increase [55]. Besides PVI non-responders, patients undergoing repeated CBA procedures [54] and patients with long SVC sleeves [26,41] or arrhythmogenic RSPV [22,34] could be optimal candidates for SVCI, especially via an empiric approach.

Empiric SVCI on top of PVI might be more effective in PAF subjects during the index procedure and more data are needed especially on contact force sensing RFA [31]. Data regarding non-RF energy sources are still in their infancy, but available studies support that CBA SVCI, especially when carried out through a non-femoral approach, is associated with complete SVCI, with an acceptable risk–benefit profile [38]. Contact force RFA is also poorly studied and could be of essence in difficult cases with an obviating SNI or PNI [53]. To date, no study has compared RFA versus CBA in the setting of SVCI, and no study of randomized design has addressed as-needed SVCI on top of PVI in PAF patients.

Studies of heterogeneous design and intent have explored both empiric and as-needed SVCI on top of PVI in PAF and peAF subjects and reported inconclusive results. These studies have failed to demonstrate any clinical benefit in terms of arrhythmia recurrence; although, safety and feasibility are largely undisputable. Mixed population demographics, a small number of participants and short follow-up are major limitations. Procedural data and safety are comparable between empiric SVCI and as-needed SVCI, and some studies underlined that empiric SVCI might be associated with reduced AF recurrences [30,33]. Since heart failure is an increasing clinical and public problem, heart failure device implantation will also increase in the forthcoming years, posing thus an urgent need to expand our safety and feasibility data on SVCI in patients with CIEDs [39].

Although many technical aspects, such as vascular access (femoral or right subclavian/jugular [38]), ultra-high resolution mapping [45], use of ICE [44], ablation energy limits [53,62], and optimal site (inside the venous or even inside posterior RA [37,47]) and approach (segmental, linear, circumferential or even a combination [32,42]) for SVCI remain challenging and unsettled, the principal question to answer is which, if any, clinical phenotype of AF patients might benefit from SVCI in the rapidly evolving era of AF catheter ablation?

## 5. Conclusions

The SVC is an important contributor to AF pathogenesis. Methodologically different studies support that SVCI is both safe and feasible. Identifying who are the best candidates and which ablation approach should to be adopted remain to be answered.

## Figures and Tables

**Table 1:** Randomized controlled trials comparing pulmonary vein isolation versus pulmonary vein isolation plus superior vena cava isolation in patients with atrial fibrillation.

Author	Ablation	N	Index	AF Type	SVC-Trigger	Female (%)	Age	HF (%)	FU	Monitoring Protocol	Post-AFCA AAD	Recurrence (%)	Complications	Feasibility	SVCI Procedural Data
Wang et al., 2008 China [28]	RF CPVI + SVC I vs. CPVI	106 (52/54)	Yes	PAF	No	45	66 ± 9	N/R	12 mo	ECG D1, W1, 1, 2, 3, 6, 9, & 12 mo, 24 h Holter ECG q 2 mo	Amio at least 1 mo	6 vs. 7 (*p* = 0.73)	2 vs. 1 FAPA 0 PNI 0 SNI 0 PVS. 0 SVCS	96%	mean SVC IT (min): 7.8 ± 2.7 mean RF app times: 6 ± 2 mean PT (min): 185.7 + 19.3 (vs. 182.7 + 17.7; *p* = 0.40) mean FT (min): 17.6 + 3.6 (vs. 16.4 + 2.7; *p* = 0.07)
Corrado et al., 2010 Italy [29]	RF CPVI + SVCI vs. CPVI	320 (160/160)	Yes	PAF, PeAF	No	26 vs. 26	55 ± 10 vs. 57 ± 9	N/R	12 mo	ECG & 48 h Holter ECG 1, 3, 6, 9, & 12 mo	No	Total 19 vs. 26 (*p* = NS); PAF 10 vs. 23 (*p* = 0.04); PeAF 20 vs. 26 (*p* = NS)	0 PNI 0 SNI 0 SVCS 1 vs. 0 PVS. 1 vs. 0 CAE 0 vs. 1 tamponade 0 vs. 1 stroke	84%	mean SVCI PT (min): 25 ± 10 mean PT (h): 3.1 ± 1.4 (vs. 2.5 ± 1.2) mean FT (min): 91 ± 27 (vs. 74 ± 23)
Da Costa et al., 2015 France [27]	RF CPVI + SVCI vs. CPVI	100 (51/49)	Yes	PAF	No	17	56 ± 9	N/R	15 ± 8 mo	24 h Holter upon symptoms & q 6 mo	at least 3 mo	12 vs. 18 (*p* = 0.6)	2 vs. 0 PNI 0 SNI 0 tamponade 0 vs. 1 stroke 0 vs. 1 PVS.	100%	mean PT (h): 2.4 ± 0.6 vs. 2.5 ± 0.8 (*p* = 0.6) mean FT (min): 14 ± 5 vs. 15 ± 6 (*p* = 0.4)

N = study population, AF = atrial fibrillation, SVC = superior vena cava, HF = heart failure, FU = follow-up period, AFCA = atrial fibrillation catheter ablation, AAD = anti-arrhythmic drug, amio = amiodarone, SCVI = SVC isolation, RF = radiofrequency, CPVI = circumferential pulmonary vein isolation, PAF = paroxysmal AF, PeAF = persistent AF, mo = months, ECG = electrocardiography, h = hours, NS = non-significant, FAPA = femoral artery pseudo-aneurysm, PNI = phrenic nerve injury, SNI = sinus node injury, PVS. = pulmonary vein stenosis, SCVS. = SVC stenosis, CAE = coronary artery embolism, SVCIT = SVC isolation time, app = application, FT = fluoroscopy time, PT = procedure time, min = minutes.

**Table 2:** Cohorts comparing different superior vena cava isolation approaches in patients with atrial fibrillation.

Author	Ablation Modality	N	Index	Previous Source	AF Type	Female (%)	Age	HF (%)	FU	Recurrence (%)	SVCI Procedural Data	Safety	Feasibility
**SVCI only**													
Chang et al., 2012 Taiwan [32]	RF SVCI vs. SVCI + CPVI	68 (37/31)	Yes	N/A	PAF	53	56 ± 12	N/R	88 ± 50 mo	higher in SVCI plus CPVI (*p* = 0.012)	N/R	0 PNI 0 SVCS 0 vs. 1 tamponade 1 vs. 0 SNI	N/R
**SVCI as-needed vs. empiric**													
Ejima et al., 2015 Japan [30]	RF CPVI + SCVI as-needed vs. empiric	186 (93/93)	Yes	N/A	PAF	29 vs. 25	58 ± 12 vs. 60 ± 10	N/R	27 ± 12 mo	44 vs. 23 (*p* = 0. 035) empiric SVCI (HR = 0.57, 95% Cl 0.318–0.999, *p* = 0.049)	mean PT (min): 208.7 ± 54 vs. 181 ± 58.3 (*p* = 0.0001) mean FT (min): 30.2 ± 15.1 vs. 16.7± 7.3 (*p* < 0.0001)	0 PNI 0 SNI 1 vs. 1 gastric hypomotility 0 vs. 1 tamponade	87% (81/93) 0 vs. 12 no SVC potentials
Zhang et al., 2020 China [33]	RF CPVI + SVCI as-needed vs. empiric	144 (72/72)	Redo	RF CPVI ± CTI	PAF	58 vs. 53	64 ± 10 vs. 64 ± 10	Excluded	19 ± 10 mo	41.7 vs. 22.2 (*p* = 0.037) empiric SVCI independent protector (OR = 0.47; 95% CI: 0.25–0.87; *p* = 0.016)	mean PT (min): 116.7 ± 32.9 vs. 123.5 ± 41.3 (*p* = 0.273) mean FT (min): 5.4 ± 2.1 vs. 5.8 ±1.8 (*p* = 0.179) mean AT (min): 13.0 ± 5.5 vs. 18.2 ± 5.2 (*p* < 0.001)	3 vs. 2 FAPA 1 vs. 0 TIA 0 vs. 1 PNI (t)	1 vs. 4 obviating PNI 0 vs. 2 no SVC potentials
**CPVI versus CPVI plus SVCI as-needed**													
Higuchi et al., 2010 Japan [26]	RF CPVI + SVCI vs. CPVI	60(12/48)	Yes (5/12 SVCI redo)	N/A	PAF, PeAF	23 (33 vs. 21)	59 ± 10 (60 ± 10 vs. 59 ± 10)	Excluded	12 mo	16.6 vs. 31.25 (2/12 vs. 15/48)	mean no of RF applications: 8 ± 2	1 PNI (t)	91.6 % (11/12)
Nakamura et al., 2016 Japan [19]	RF CPVI + SVCI vs. CPVI	36 (7/29)	Redo	RF (CPVI + CTI)	PAF, PeAF	14 (14 vs. 14)	59 ± 9 (58 ± 10 vs. 59 ± 10)	Excluded	1538 ± 426 d	0 vs. 27.6	N/R	Zero	71.42 % (2 obviating PNI)
Takigawa et al., 2017 Japan [11]	RF CPVI ± CTI vs. CPVI + SCVI ± CTI vs. CPVI + non PV foci: Linear ablations (LA roof ± bottom ± MAI ± CTI)	865 (740/57/68)	Yes	N/A	PAF	23 (20/40/31)	61 ± 10 (61 ± 10/60 ± 10/61 ± 11)	5.8 (5.1/3.5/14.7)	54 ± 39 mo	40.3 vs. 38.6 vs. 54.4 (*p* = 0.03)	N/R	5.9% 5.3% 4.2% (*p* = NS)	N/R
Yoshida et al., 2017 Japan [34]	RF CPVI + SVCI vs. CPVI	121 (22/99)	Yes	N/A	PAF	18 (36 vs. 14)	63 ± 9 (64 ± 9 vs. 63 ± 9)	N/R	22 mo	N/R Redo ablation 1.68 ± 0.78 vs. 1.28 ± 0.45 procedures (*p* = 0.002)	N/R	N/R	100%
**CPVI versus CPVI plus SVCI empiric**													
Yoshiga et al., 2018 Japan [35]	RF CPVI vs. SVCI + CPVI	70 (55/15)	redo	N/A	PeAF	23	61 ± 12	17.1	32 (12–57)	34.3 vs. 25.7	N/R	1 stroke 1 abdominal bleeding	N/R
Jin et al., 2019 Republic of Korea [36]	RF CPVI + CTI vs. CPVI + CTI + SCVI	614 (307/307)	Yes	N/A	PAF, PeAF	27	58 ± 11	5.2	41 ± 24 mo	46.3 vs. 26.1 (*p* < 0.001), independent protector (HR = 0.59; 95% CI, 0.44–0.78; *p* < 0.001)	mean PT(min): 165 ± 44 vs. 184 ± 34 (*p* < 0.001) mean AT (s): 3996 ± 1220 vs. 4809 ± 1046 (*p* < 0.001)	0 PNI 0 vs. 1AVB 5 vs. 6 Tamponade	N/R
Knecht et al., 2022 Switzerland [37]	RF CPVI + SVCI vs. CPVI	344 (75/269)	redo	PVI (CB 76% RF 24%)	PAF, PeAF	27	60 ± 10	N/R	320 d median	27 vs. 20 (*p* = 0.151) (SVCI HR = 1.3 95% CI:0.836–2.022; *p* = 0.244	mean PT (min): 99 ± 34 vs. 110 ± 42 (*p* = 0.063) mean FT (min): 3 ± 6 vs. 5 ± 7 (*p* = 0.048) mean total RF (s): 779 ± 416 vs. 859 ± 491 (*p* = 0.244) mean SVCIT (s): 265 ± 196	0 PNI	96% (72/76) (3 obviating PNI)
Overeinder et al., 2021 Belgium [38]	CB PVI + SVCI vs. PVI	100 (50/50)	Yes	N/A	PAF	34 vs. 30	55 ± 12 56 ± 12	10 vs. 22	12 mo	10 vs. 28 (HR = 0.78; 95% CI: 0.64–0.89; *p* = 0.04)	mean SVCIT (s): 36.7 ± 29 mean SVC FT (min): 1.6 ± 0.8 mean PT (min): 88.7 ± 13.6 vs. 70.1 ± 15.2; *p* < 0.001 mean FT (min): 25.1 ± 8.4 vs. 22.9 ± 12; *p* = 0.29	2 PNI (t) 0 SNI 0 vascular	94% (3 obviating PNI)
**Other**													
Simu et al., 2022 Germany [39]	RF HPSD + SVCI ± LA substrate vs. HPSD ± LA substrate	276(128/148)	redo	N/R	PAF, PeAF	45	67 ± 10	N/R	12 mo	ER: 19 vs. 15 (*p* = 0.304) LR: 27 vs. 26 (*p* = 0.853) SVCI (HR = 0.951; 95% CI: 0.558–1.621; *p* = 0.853)	mean PT (min): 84.2 ± 26.6 vs. 86.4 ± 27.6 (*p* = 0.503) mean FT (min): 7.4 ± 4.8 vs. 7.9 ± 5.8 (*p* = 0.505) mean AT (min): 13.8 ± 7.2 vs. 14.1 ± 9.4 (*p* = 0.784)	0 PNI 0 SNI 0 SVCS 0 tamponade	N/R
Kataoka et al., 2020 Japan [40]	RF CPVI + SVCI ± CTI; CIED vs. non-CIED	34/34 (age-sex-AF matched)	yes	N/A	PAF, PeAF	35 vs. 29	58 ± 13 vs. 59 ± 12	N/R	22 mo	35.3 vs. 29.4 (*p* = 0.6)	mean PT (min): 175.3 ± 12.4 vs. 152.6 ± 12.6 (*p* = 0.204) mean FT (min): 15.8 ± 1.9 vs. 10.5 ± 1.8 (*p* = 0.046) mean SVCIT (min): 6.3 ± 4.2 vs. 6.3 ± 5.1 (*p* = 0.99)	0 PNI 3 lead failures (8.8%)	91.2% vs. 100% (*p* = 0.07)

N = study population, Index = first ablation procedure, redo = repeat ablation procedure due to arrhythmia recurrence, AF = atrial fibrillation, SVC = superior vena cava, HF = heart failure, FU = follow-up period, SCVI = SVC isolation, RF = radiofrequency, CB = cryoablation, CPVI = circumferential pulmonary vein isolation, CTI = cavotricuspid isthmus, MAI = mitral annulus isthmus, HPSD = high power short duration, CIED = cardiac implantable electronic device, PAF = paroxysmal AF, PeAF = persistent AF, mo = months, h = hours, NS= non-significant, ER = early arrhythmia recurrence, LR = late arrhythmia recurrence, FAPA = femoral artery pseudo-aneurysm, PNI = phrenic nerve injury, SNI = sinus node injury, PVS. = pulmonary vein stenosis, SCVS. = SVC stenosis, TIA = transient ischemic attack, (t) = transient, AVB = atrioventricular block, SVCIT = SVC isolation time, app = application, FT = fluoroscopy time, PT = procedure time, AT = ablation time, min = minutes, N/A = not applicable, N/R = not reported., OR = odds ratio, CI = confidence intervals, HR = hazard ratio.

## Data Availability

Upon reasonable request.

## Supplementary Materials

The following supporting information can be downloaded at https://www.mdpi.com/article/10.3390/biomedicines11041022/s1.
