# Supplementary material for: Efficacy, Safety and Feasibility of Superior Vena Cava Isolation in Patients Undergoing Atrial Fibrillation Catheter Ablation: An Up-to-Date Review
Source: Biomedicines. 2023 Mar 27;11(4):1022. doi: 10.3390/biomedicines11041022 (PMC10136264; doi:10.3390/biomedicines11041022)
Supplement: Supplementary file 1 [file biomedicines-11-01022-s001.zip › biomedicines-2272416-supplementary.pdf]

Supplementary Table S1: Cohorts reporting on superior vena cava isolation in patients with atrial fibrillation

**Author**	**Energy source**	**N**	**Index**	**Previous Source**	**AF type**	**SVC-trigger**	**Female (%)**	**Age**	**HF (%)**	**FU**	**Recurrence (%)**	**Safety**	**Feasibility**	**SVCI Procedural data**
**CPVI+ as needed SVCI**														
Tsai et al 2000 Taiwan	RF (CPVI +SVCI)	8	Yes	N/A	PAF	yes (8)	50	55 ± 12	N/R	9±3 mo	(0/8)	0 SVCS	75% (6/8) 2 partly eliminated	mean RF apps: 5±3
Arruda et al 2008 USA	RF	24	Yes	N/A	PAF, PeAF	yes	N/R	N/R	N/R	450 ± 180 d	0	0 SVCS 0 SNI	N/R	N/R
Chen et al 2011 China	RF, CPVI + additional linear ablation (MAI, CTI, roof line & CS) ± SVCI	132	Yes	N/A	AF or atypica l Flutter	35/132	50 (3/6)	N/R	excluded	N/R	N/R	6/132 SNI	N/R	N/R
Miyazaki et al 2014 Japan	RF (CPVI ± SVCI)± CFAEs/CTI	836 44/792	Yes	N/A	PAF, PeAF	yes	30 vs. 24	59 ± 11 vs.63 ± 10	N/R	17 mo (9-27)	11.4 1.5 ± 0.7 procedures	2 PNI (t)	100	N/R
Shi et al 2015 China	RF (CPVI+SVCI)± LA bottom line, MAI, CTI	30	Yes	N/A	PAF	yes	40	59 ± 16	N/R	18 ± 6 mo	6.6 (2) ER 3.3(1) LR	0 PNI 0 SNI 0 tamponade	70%	mean SVCIT (min): 14.3 ± 11.5 mean SVCI FT (min): 9.6 ± 3.8 mean RF apps: 11.2 ± 3.1
Xu et al 2017 China	RF (CPVI± CFAE,LA roof, AT ablation)±SVCI	102	Yes	N/A	PeAF	1/102	37	63 ± 9	8	12 mo	56.9	Zero	N/R	mean PT (min): 166±22 mean FT (min): 17±3
Tanaka et al 2018 Japan	RF [CPVI±Linear ablations (LA roof± bottom ± MAI])±CTI	113	Yes	N/A	PAF, PeAF	38.1 % (43/11 3)	28	63 ±12	7	N/R	N/R	2 PNI 0SNI	N/R	mean SVC mapping: 10.8±5.5 min mean RF app: 4.2±0.9 vs.. 10.2±2.8; p<0.0001 mean RF energy (J) 5,042±1,581 vs.. 10,582±2,335, p<0.000 mean SVCIT (min) 3.3±1.2 vs.. 10.2±2.8 p<0.0001

Miyazaki et al 2019 Japan	sg-CB (CPVI + RF SVCI)	34 (18)	Yes	N/A	PAF, PeAF	yes	29	64 ± 11	N/R	N/R	N/R	0 PNI	100	mean RF apps: 11.7 ± 5.9<br>mean RF (min): 4.1 ± 2.2<br>mean SVCIT (min): 9.9 ± 8.9
Wei et al 2020 China	CB-2ndg (PVI+SVCI)	26	Yes	N/A	PAF	yes	50	60 ± 8	N/R	13 ± 6 mo	15.4	5 PNI (t)<br>2 SNI (t)<br>0 SVCS	80.8%	Mean PT (min): 61.6 ± 15.3<br>Mean FT (min): 16.3 ± 9.9<br>Mean no of freeze :2.1 ± 1.1<br>Mean SVCIT (sec): 22.5 ± 14.2<br>Mean AT (sec): 94.5 ± 22.3
Nishiyama et al 2020 Japan	RF (CPVI + SVCI)	15	Yes	N/A	N/R	yes	27	59 ± 10	N/R	3 ± 0.4 y	13.3	0 PNI	100	mean RF apps (SVC): 7.0 ± 5.1<br>mean SVCIT(sec): 176 ± 125
Nyuta et al 2021 Japan	RF or CB (CPVI±SVCI)± LA roof and bottom lines, CTI	427 143/284	Yes	N/A	PAF, PeAF	yes	34 (29 vs. 37)	72 ± 9 (71 ± 10 vs. 72 ± 9)	N/R	12 mo	PAF 14<br>PeAF 21<br>L-PeAF 38	Total:<br>1 PNI(t)<br>5 tamponade<br>2pericarditis<br>1 stroke<br>2 hematomas	83% (24 obviating PNI)	N/R
**Redo subgroup**														
Inada et al 2015 Japan	RF (CPVI+ CTI±SVCI)±substrate	336 (43)	yes or redo	N/R	PAF	yes	18 (21 vs. 17)	56 ± 11 (57 ± 11 vs. 56 ± 11)	N/R	N/R	20	2 PNI (t)<br>1 SNI (t)	93 % (3/43 obviating PNI)	N/R
Miyazaki et al 2016 Japan	RF or sg-CB (CPVI+SVCI) under right diaphragmatic CMAPs	39	yes (5 redo)	N/R	PAF	yes	26	63 ± 10	N/R	N/R	N/R	1 PNI (PVI)	100	mean RF duration (SVC) (min): 4.6 ± 1.7 min<br>mean RF apps: 9.4 ± 3.3
Miyazaki et al 2013 Japan	RF CPVI ± CFAE	76	Redo	RF CPVI+ SVCI	PAF, PeAF	yes	23	65 ± 9	N/R	16 ±16 mo	12.5<br>Second redo	Total:<br>4 PNI (t)	Redo: 96% (54/56) first procedure: 82.9%	Redo: 4.2±2.9<br>Total: mean RF apps: 7.3±3.1
Takamiya et al 2021 Japan	RF (CPVI±SVCI))± LA roof and bottom lines, CTI, non-PV foci, CFAE	119	Redo	sg-CB-PVI±CTI non-PV triggers	PAF	yes (31) or SVC sleeve longer than 30 mm	60	66 ± 10	N/R	461d (376–739)	total: 21	Total:<br>1 SNI<br>2tamponade<br>1 hematoma	100 % (31/31)	N/R
**CPVI+ empiric SVCI**														

Arruda et al 2008 USA	RF	217	Yes	N/A	PAF, PeAF	No	N/R	N/R	N/R	450 ± 180 d	N/R	0 SVCS<br>0 SNI<br>2 PNI (t)	82%<br>40 patients (18%) PNI at risk	N/R
Gianni et al 2017 Italy	RF CPVI+ SVCI	105	Yes or redo	N/A	PAF, PeAF	No	18	67 ± 9	N/R	9 ± 6mo	N/R	2 PNI<br>0 SNI	98%<br>(2 obviating PNI)	Mean PT (min): 125± 37<br>Median SVCI RF time (sec) : 134 (89–203)
Arceluz et al 2018 Spain	LB (CPVI±SVCI)	13	Yes	N/A	PAF, PeAF	No	15	59 ± 11	30.7	19 ± 3 mo	15	3 PNI (p)<br>(23%)	61% (8/13)<br>(3 PNI<br>1 laser malfunction<br>1 lead failure)	mean SVCI power (W) : 6.3 ± 2.3<br>mean SVCIT (min): 5.38 ± 2.4
Rubio Campal et al 2020 Spain	CB (PVI+SVCI)	30	yes or redo	N/A	PAF, PeAF	No	26	62 ± 9	N/R	5 ± 2 mo	11	1 PNI (t)<br>1 SNI (t)<br>0 SVCS	89%<br>(2 no SVC potential)	mean cryo time (sec): 92 ± 15<br>Mean no of freeze: 2.3 ± 1<br>Mean SVCIT (sec): 37 ± 20<br>mean FT (min); PV 17 ± 2<br>mean FT (min); SVC 1.5 ± 0.9
Omuro et al 2021 Japan	RF (CPVI+ SVCI vs. CPVI)	153<br>102/51	Yes	N/A	NPAF	No	22	65 ± 10	26.1	18 mo	N/R	0 vs. 1 vascular<br>0 PNI	100%	N/R
Gu et al 2021 China	RF(non-PV foci, SVCI, CFAE, linear ablation)	64	redo	RF PVI	PAF, PeAF	no	45	61 ± 11	N/R	20 ± 10 mo	21 vs. 50 independent protector (HR: 0.338; 95% CI: 0.131–0.873; p = 0.025)	N/R	N/R	N/R
**Mixed modalities and index procedures**														
Kimura et al 2012 Japan	RF (CPVI± linear, CFAE)	41	Yes or redo	N/R	PAF, PeAF	(yes if index, no in redo)	19	59 ± 9	N/R	12 ± 7 mo	12.2	1 PNI (t)	100%	mean RF (sec): 365 ± 204<br>No of SVCI app times: 12 ± 6<br>mean RF power 20± 5 W
Fukumoto et al 2014 Japan	RF (CPVI + SVCI)	25	yes or redo	N/R	PAF, PeAF	yes (13) empiric (12)	24	57 ± 9	N/R	619 ± 5d	4 (1/25)	1 PNI (t)	100%	Mean RF apps: 10 ± 6.5

N=study population, Index= first ablation procedure, redo= repeat ablation procedure due to arrhythmia recurrence, AF=atrial fibrillation, SVC=superior vena cava, HF=heart failure, FU= follow up period, SCVI= SVC isolation, RF= radiofrequency, CB= cryoablation, LB= laser ablation, CPVI= circumferential pulmonary vein isolation, CTI= cavotricuspid isthmus, MAI= mitral annulus isthmus, CFAE=complex fractionated atrial electrograms, CMAPs= compound motor action potentials, PAF=paroxysmal AF, PeAF= persistent AF, L-PeAF= long standing PeAF, mo= months, h=hours, NS= non-significant, ER= early arrhythmia recurrence, LR= late arrhythmia recurrence, PNI=phrenic nerve injury, SNI= sinus node injury, PVS.= pulmonary vein stenosis, SCVS.= SVC stenosis, (p)=permanent, (t)= transient, SVCIT= SVC isolation time, app=application, FT= fluoroscopy time, PT= procedure time, AT=ablation time, min= minutes, N/A= not applicable, N/R= not reported.
